# Minieditorial: Competência Prognóstica Distinta entre Modelo Clínico e Anatômico nas Síndromes Coronarianas Agudas: Comparação por Tipo de Desfecho

**DOI:** 10.36660/abc.20200758

**Published:** 2020-08-19

**Authors:** Roberto Coury Pedrosa

**Affiliations:** Departamento de Cardiologia Hospital Universitário Clementino Fraga Filho Brasil Departamento de Cardiologia, Hospital Universitário Clementino Fraga Filho / Instituto do Coração Edson Saad - Universidade Federal do Rio de Janeiro, Rio de Janeiro, RJ - Brasil; 1 Instituto do Coração Edson Saad Universidade Federal do Rio de Janeiro Rio de Janeiro RJ Brasil

**Keywords:** Doenças Cardiovasculares/mortalidade, Infarto do Miocárdio, Síndrome Coronariana Aguda, Saúde Pública, Organização e Administração, Cuidados Médicos, Equipamentos para Diagnóstico, Medição de Risco, Intervenção Coronária Percutânea

Eventos cardiovasculares, principalmente o infarto agudo do miocárdio, são as principais causas de morte no Brasil. Em alguns países europeus (por exemplo, França, Portugal, Itália), as taxas de mortalidade em 30 dias por infarto diminuíram nas últimas décadas, chegando a valores tão baixos quanto 3% a 5%. Isso reflete a organização da logística da saúde, incluindo atendimento pré-hospitalar, protocolos unificados, treinamento, regulação central e compromisso com o atendimento.^1^ No Brasil, as taxas de mortalidade em 30 dias variam de 3% a 5% em centros avançados e 30% naqueles centros nos quais o atendimento não aplica as diretrizes recomendadas.^2^ Essa disparidade geralmente reflete um sistema de saúde pública ainda deficiente que carece de fluxogramas diagnósticos, protocolos institucionais, regulação central ou profissionais capazes de interpretar o diagnóstico de infarto utilizando um eletrocardiograma (ECG). Infelizmente, ainda verificamos que muitos centros carecem de equipamentos nos setores de emergência (desfibrilador, materiais de intubação, ventiladores, eletrocardiografias, drogas vasoativas, monitores cardíacos, marcapassos temporários e drogas fibrinolíticas) e unidades coronarianas, além da falta de profissionais qualificados para fornecer o melhor tratamento.

O reconhecimento de que as síndromes coronarianas agudas (SCA) constituem uma doença heterogênea em relação ao prognóstico foi fundamental para a correta identificação de indivíduos de maior risco, que necessitam de intervenção mais intensiva.^3^ Foi demonstrado que nem todos os pacientes com SCA pertencem a categorias de risco alto ou muito alto; há uma porcentagem considerável composta por pacientes jovens sem fatores de risco clássicos que podem ser adequadamente classificados em categorias de risco intermediário ou mesmo baixo. Portanto, é importante identificar os pacientes de maior risco que necessitam de tratamento mais intensivo. Estudos clínicos importantes contribuíram para a evolução da abordagem em pacientes com SCA.^4 - 7^ Atualmente, o foco emergente no envolvimento de fatores socioeconômicos no risco cardiovascular tem sido constantemente relatado.^8^

Nesta edição, Viana et al.^9^ apresentaram um estudo que comparou a capacidade preditiva dos escores SYNTAX^10^ e GRACE^11^ em predizer resultados isquêmicos não-fatais (infarto ou angina refratária) e morte cardiovascular durante a hospitalização de pacientes com SCA. Eles chamam atenção para o fato de que eventos isquêmicos não-fatais recorrentes representam o fenômeno de instabilidade da placa aterosclerótica, enquanto a morte após um evento isquêmico resulta da gravidade do insulto e da resistência do paciente. A natureza fisiopatológica diferente desses tipos de eventos pode fazer com que os dados clínicos e anatômicos tenham capacidades prognósticas diferentes, dependendo do tipo de desfecho. Se isso for verdade, a generalização do valor prognóstico em relação aos “desfechos cardiovasculares” seria comprometida, tornando necessário individualizar a previsão de cada modelo para o tipo de desfecho. Considerando a justificativa da escassez de estudos dessa natureza relatados na literatura com o objetivo de responder a essa pergunta, os autores realizaram um estudo serial de casos consecutivos entre 2007 e 2014 em um hospital terciário no Brasil, com o objetivo de avaliar e comparar o valor prognóstico de dados clínicos e anatômicos em relação a resultados fatais e não-fatais em pacientes com SCA. Os pacientes admitidos consecutivamente com SCA submetidos à angiografia coronariana foram recrutados. O escore SYNTAX foi utilizado como modelo anatômico e o escore GRACE como modelo clínico. Dos 365 pacientes analisados, a média de idade foi de 64 ± 14 anos, sendo 58% do sexo masculino, 19% dos quais com infarto do miocárdio com supradesnivelamento do segmento ST. Doença coronariana triarterial ou doença da coronária esquerda estava presente em 36% da amostra.

A mediana do escore SYNTAX foi 9 (IIQ = 2,5–20) e a mediana do escore GRACE foi de 117 (IIQ = 90–144). Ao analisar os tercis de risco previstos pelo escore SYNTAX, 81% dos pacientes apresentaram valor baixo (0 a 22), 10% apresentaram valor intermediário (23 a 32) e apenas 8,5% apresentaram valor alto (≥ 33). Em relação ao escore GRACE, 44% apresentaram baixo risco (<109), 28% risco intermediário (110 a 139) e 29% alto risco (≥ 140). Entre os 365 indivíduos, houve uma incidência de 4,4% de óbito hospitalar e 11% de desfechos isquêmicos não-fatais. Para morte cardiovascular, ambos os escores - SYNTAX e GRACE - apresentaram capacidade discriminatória, com estatística-C semelhante: 0,80 (IC95%: 0,70-0,92) e 0,89 (IC95% 0,81-0,96), respectivamente, com p = 0,19. Quanto aos desfechos isquêmicos não-fatais, o escore SYNTAX apresentou um valor preditivo (estatística-C = 0,64; IC 95% 0,55-0,73); entretanto, o escore GRACE não mostrou associação com esse tipo de desfecho (estatística-C) = 0,50; IC95%: 0,40-0,61), com p = 0,027.

Em conclusão, os autores propõem um refinamento adicional na previsão de risco em pacientes com síndrome coronariana aguda (SCA). Tanto o paradigma clínico (GRACE) quanto o anatômico (SYNTAX) demonstraram ter uma boa capacidade preditiva de morte. Entretanto, apenas o modelo anatômico foi capaz de prever eventos não-fatais recorrentes. Essa demonstração de que os escores comumente utilizados no manejo clínico de pacientes com SCA pode ter predileção por desfechos diferentes não foi descrita na literatura até o momento.

No entanto, a interpretação desses resultados deve considerar a presença de um erro do tipo β nesta análise, devido ao pequeno número de pacientes com eventos de morte (19 óbitos em 365 pacientes). Os fatores de confusão não-controlados e a incerteza introduzida por uma grande diferença no terceiro tercil foram observados em ambos os escores para o evento de morte. O desenho do presente estudo (série de casos), com coleta retrospectiva de dados, representa uma amostra parcial da população de pacientes hospitalizados por SCA, compreendendo uma subpopulação submetida à angiografia coronariana, não representando, portanto, o cenário real da população de SCA atendida em hospital, mas um subgrupo selecionado com o melhor prognóstico para o qual o médico assistente indicou a realização de uma angiografia coronariana. A análise dos dados foi realizada com base em relatos, e os dados de imagem não foram obtidos pela revisão das imagens e dos achados. Por fim, estudou-se uma população em diferentes estágios evolutivos pós-SCA, sendo adotado o exame de angiografia coronariana como referência.

Da mesma forma, reconhecemos que, apesar de sua utilidade, os escores de risco tendem a superestimar o mesmo,^12^ além de apresentar poder limitado de discriminação entre indivíduos de alto e baixo risco. Eles superestimam o risco porque às vezes são derivados da população em geral e outras vezes de populações específicas. Há uma dificuldade em estratificar o risco por meio de escores, uma vez que a maioria dos eventos continua ocorrendo em pacientes considerados de baixo risco ou risco intermediário. As limitações dos escores de risco resultam da fisiopatologia da própria SCA. Estudos de randomização mendeliana, estudos de coorte longitudinal com populações jovens, além de estudos realizados em autópsias, demonstraram que a exposição ao risco de aterosclerose ocorre precocemente, varia em intensidade ao longo da vida e inclui fatores genéticos e ambientais não considerados nos escores. Uma única medida de fatores de risco no indivíduo adulto com SCA falha na quantificação da exposição ao risco dependente do tempo. O risco da doença seria expresso com mais precisão, devido à exposição cumulativa a todos esses determinantes de risco ao longo da vida.^13^

É importante observar que, no processo dinâmico do estabelecimento do risco no paciente no qual há suspeita e / ou confirmação de SCA, os critérios clínicos são de suma importância, conseguindo identificar, sem qualquer outro recurso, os pacientes de maior risco para a ocorrência de morte ou eventos isquêmicos recorrentes. A avaliação clínica continuada é sempre essencial, seja devido a complicações abruptas que exigem mudanças rápidas de conduta ou à necessidade de critérios clínicos ajustados ao caso. A criação e atualização da avaliação de variáveis clínicas que podem predizer o risco de resultados adversos em momentos bem definidos no tempo são necessárias, principalmente em termos de custo-efetividade.

Utilizando apenas elementos clínicos, podemos definir pacientes de alto risco para eventos cardíacos maiores, tanto a curto quanto a longo prazo, pelas características de seus sintomas, histórico pessoal e exame físico. No entanto, quatro variáveis sempre parecem significativas ao tentar prever a morte após SCA. Variáveis clínicas: idade, disfunção renal (expressa pela creatinina sérica), histórico de IAM prévio, diabetes mellitus - que indica uma disfunção fisiológica global caracterizada por glicemia elevada - e os dados de disfunção ventricular esquerda.

Portanto, na presença de SCA, na maioria dos casos, é a suspeita clínica inicial que fornece a melhor terapia e o prognóstico. Nas atuais condições socioeconômicas, a avaliação na chegada do paciente ao hospital é a que apresenta maior possibilidade de efetividade junto à doença.

Novamente, enfatizamos que esses “escores” devem servir como guias, e não como “amarras”, para o nosso julgamento clínico, com o último sendo capaz de fazer uso das informações existentes para escolher a melhor alternativa para o paciente. Esses escores devem estar abertos a interpretações e opções de tratamento que podem ser limitadas por recursos financeiros. Um diagnóstico precoce, juntamente com um bom tratamento e reabilitação cardíaca, promove uma boa recuperação do paciente.

Em nosso contexto, mudanças na melhora organizacional, bem como na educação do paciente, profissionais do atendimento de emergência e coordenação com agentes do sistema de saúde público ou privado resultarão em uma diminuição significativa da mortalidade por SCA.
