# Supplementary material for: Minieditorial: Competência Prognóstica Distinta entre Modelo Clínico e Anatômico nas Síndromes Coronarianas Agudas: Comparação por Tipo de Desfecho
Source: Arq Bras Cardiol. 2020 Aug 19;115(2):226–8. [Article in Portuguese] doi: 10.36660/abc.20200758 (PMC8384282; doi:10.36660/abc.20200758)
Supplement: Translation

# Translation: Short Editorial: Distinct Prognostic Competence between the Clinical and Anatomical Models in Acute Coronary Syndromes: Comparison by Type of Outcome

**Authors:** Roberto Coury Pedrosa

**Affiliations:** 1 Brazil Departamento de Cardiologia, Hospital Universitário Clementino Fraga Filho / Instituto do Coração Edson Saad - Universidade Federal do Rio de Janeiro, Rio de Janeiro, RJ - Brazil

**Keywords:** Cardiovascular Diseases/mortality, Myocardial Infarction, Acute Coronary Syndrome, Public Health, Organizationa and Administration, Diagnostic Equipment, Risk Assessment, Percutaneous Coronary Intervention

Cardiovascular events, especially acute myocardial infarction, are the main causes of death in Brazil. In some European countries (e.g., France, Portugal, Italy), the 30-day mortality rates due to infarction have declined in recent decades to as low as 3% to 5%. This reflects the organization of healthcare logistics, including pre-hospital care, unified protocols, training, central regulation, and commitment to care.^1^ In Brazil, the 30-day mortality rates vary from 3% to 5% in advanced centers and 30% in those where care does not apply the recommended guidelines.^2^ This disparity usually reflects a still deficient public healthcare system that lacks diagnostic flowcharts, institutional protocols, central regulation, or professionals who are able to interpret the diagnosis for infarction using an electrocardiogram (ECG). Unfortunately, we still find that many centers lack equipment in emergency sectors (defibrillator, intubation materials, ventilators, electrocardiographs, vasoactive drugs, cardiac monitors, temporary pacemakers, and fibrinolytic drugs) and coronary units, as well as the lack of qualified professionals to provide the best treatment.

The recognition that acute coronary syndromes (ACS) constitute a heterogeneous disease in relation to prognosis was fundamental for the correct identification of individuals at higher risk, who require more intensive intervention.^3^ It has been shown that not all patients with ACS belong to high or very high risk categories; there is a considerable percentage consisting of young patients without classic risk factors that can be adequately classified into intermediate or even low risk categories. Thus, it is important to identify those patients at higher risk who need more intensive treatment. Important clinical studies have contributed to the evolution of the approach to patients with ACS.^4 - 7^ Currently, the emerging focus on the involvement of socioeconomic factors in cardiovascular risk has been constantly reported.^8^

In this edition, Viana et al.^9^ presented a study that compared the predictive capacity of the SYNTAX^10^ and GRACE^11^ scores for the prediction of non-fatal ischemic outcomes (infarction or refractory angina) and cardiovascular death during hospitalization of patients with ACS. They draw attention to the fact that recurrent non-fatal ischemic events represent the phenomenon of atherosclerotic plaque instability, while death after an ischemic event results from the severity of the insult and the patient’s resistance. The different pathophysiological nature of these types of events can cause the clinical and anatomical data to have different prognostic capacities, depending on the type of outcome. If this is true, the generalization of the prognostic value in relation to the “cardiovascular outcomes” would be compromised, making it necessary to individualize the prediction of each model for the type of outcome. Considering the justification of the scarcity of studies of this nature reported in the literature aimed at answering this question, the authors performed a serial study of consecutive cases between 2007 and 2014 in a tertiary hospital in Brazil, with objective of evaluating and comparing the prognostic value of clinical and anatomical data in relation to fatal and non-fatal outcomes in patients with ACS. Patients consecutively admitted with ACS submitted to coronary angiography were recruited. The SYNTAX score was used as an anatomical model and the GRACE score as a clinical model. Of the 365 analyzed patients, the mean age was 64 ± 14 years, with 58% male individuals, 19% of whom had ST-segment elevation myocardial infarction. Coronary heart disease with triple vessel or left main coronary artery disease was present in 36% of the sample.

The median SYNTAX score was 9 (IQR = 2.5–20) and the median GRACE score was 117 (IQR = 90–144). When analyzing the risk tertiles predicted by the SYNTAX score, 81% of the patients had a low value (0 to 22), 10% showed an intermediate value (23 to 32) and only 8.5% had a high value (≥ 33). Regarding the GRACE score, 44% showed low risk (<109), 28% intermediate risk (110 to 139) and 29% high risk (≥ 140). Among the 365 individuals, there was an incidence of 4.4% of hospital death and 11% of non-fatal ischemic outcomes. For cardiovascular death, both scores - SYNTAX and GRACE - showed discriminatory capacity, with similar C-statistics: 0.80 (95% CI: 0.70–0.92) and 0.89 (95% CI 0.81– 0.96), respectively, with p = 0.19. As for non-fatal ischemic outcomes, the SYNTAX score had a predictive value (C-statistic = 0.64; 95% CI 0.55–0.73); however, the GRACE score did not show an association with this type of outcome (C-statistic) = 0.50; 95% CI: 0.40–0.61), with p = 0.027. In conclusion, the authors propose a further refinement in risk prediction in patients with acute coronary syndrome (ACS). Both the clinical paradigm (GRACE) and the anatomical one (SYNTAX) were shown to have a good predictive capacity for death. However, only the anatomical model was able to predict recurrent non-fatal events. This demonstration that the scores commonly used in the clinical management of patients with ACS may have a predilection for different outcomes has not been described in the literature to date.

Nevertheless, the interpretation of these results should consider the presence of a type β error in this analysis, because of the small number of patients with death events (19 deaths in 365 patients). The non-controlled confounding factors and the uncertainty introduced by a large difference in the third tertile were observed in both scores for the death event. The design of the present study (case series), with a retrospective data collection, represents a partial sample of the population of patients hospitalized for ACS, comprising a subpopulation submitted to coronary angiography, therefore not representing the real scenario of the ACS population treated in this hospital, but a selected subgroup with the best prognosis for which the attending physician indicated the performance of a coronary angiography. Data analysis was performed based on reports, and the imaging data were not obtained by reviewing the images and findings. Finally, a population in different post-ACS evolutionary stages was studied, as the coronary angiography exam was adopted as the reference.

Likewise, we recognize that, despite their usefulness, risk scores tend to overestimate risk,^12^ besides presenting limited power of discrimination between high and low risk individuals. They overestimate risk because they are sometimes derived from the general population, and sometimes from specific populations. There is a difficulty in stratifying risk by means of scores, since most events continue to occur in patients considered to be at low or intermediate risk. The limitations of risk scores result from the pathophysiology of ACS itself. Mendelian randomization studies, longitudinal cohort studies with young populations, in addition to autopsy studies, demonstrated that exposure to the risk of atherosclerosis occurs early, varies in intensity throughout life and includes genetic and environmental factors not taken into account in the scores. A single measure of risk factors in the adult individual with ACS fails to quantify the exposure to time-dependent risk. The risk of disease would be expressed more precisely, due to the cumulative exposure to all these risk determinants throughout life.^13^

It is important to note that, in the dynamic process of establishing risk in the patient in which ACS is suspected and / or confirmed, clinical criteria are of paramount importance, managing to identify, without any other resource, the patients at highest risk for the occurrence of death or recurrent ischemic events. Continued clinical evaluation is always essential, whether due to abrupt complications that require rapid changes in conduct or the need for clinical criteria adjusted to the case. The formulation and updating of the evaluation of clinical variables that can predict the risk of adverse results at well-defined points in time are necessary, particularly in terms of cost-effectiveness.

Using only clinical elements, we can define high-risk patients for major cardiac events, both in the short and long term, by the characteristics of their symptoms, their personal history and physical examination. Nevertheless, four variables always seem significant when trying to predict death after ACS. Clinical variables: age, renal dysfunction (expressed by serum creatinine), history of previous AMI, diabetes mellitus - which indicates a global physiological dysfunction characterized by elevated blood glucose - and the left ventricular dysfunction data.

Therefore, in the presence of ACS, in most cases, it is the initial clinical suspicion that provides the offer of the best therapy as well as the prognosis. In the current socio-economic conditions, the evaluation at the patient’s arrival at the hospital is the one that ultimately has the greatest possibility of effectiveness along with the disease.

Again, we emphasize that these “scores” should serve as guides, and not as ‘strings’ for our clinical judgment, with the latter being able to make use of the existing information to choose the best alternative for the patient. Such scores must be open to interpretations and treatment options that may be limited by financial resources. An early diagnosis together with good treatment and cardiac rehabilitation promote good patient recovery.

In our context, changes in the organizational improvement, as well as in patient education, professionals in the emergency department, and coordination with agents in the public or private health system will result in a significant decrease in mortality due to ACS.
